# Cytomegalovirus IL‐10 in Plasma as a Marker of Active Infection in Allogeneic Hematopoietic Transplant Recipients: An Exploratory Study

**DOI:** 10.1002/jmv.70806

**Published:** 2026-01-13

**Authors:** Ángela Sánchez‐Simarro, Eliseo Albert, Estela Giménez, Ester Colomer, Ariadna Pérez, José Luis Piñana, Carlos Solano, David Navarro

**Affiliations:** ^1^ Microbiology Service Clinic University Hospital, INCLIVA Health Research Institute Valencia Spain; ^2^ CIBER de Enfermedades Infecciosas, Instituto de Salud Carlos III Madrid Spain; ^3^ Hematology Service Clinic University Hospital, INCLIVA Health Research Institute Valencia Spain; ^4^ Department of Medicine School of Medicine University of Valencia Valencia Spain; ^5^ Department of Microbiology School of Medicine University of Valencia Valencia Spain

**Keywords:** active CMV infection, allogeneic hematopoietic stem cell transplantation, CMV DNAemia, cmvIL‐10, cytomegalovirus (CMV)

## Abstract

We investigated whether plasma cytomegalovirus (CMV) IL‐10 (cmvIL‐10) levels could serve as a biomarker of active CMV replication in allogeneic hematopoietic transplant recipients (allo‐HCT) in the presence or absence of letermovir (LMV) prophylaxis. A total of 189 leftover plasma samples that tested positive for CMV DNA (Alinity *m* CMV assay), representing 33 episodes of CMV DNAemia were run on a laboratory‐developed enzyme‐linked immunosorbent assay for cmvIL‐10 quantification. Eighteen episodes developed during LMV prophylaxis. Overall, 16 episodes of CMV DNAemia were classified as clinically significant (CsCMVi). There was an overall very weak correlation between the two biomarkers (Rho = 0.10; *p* = 0.16). Overall, the median cmvIL‐10 area under the curve (AUC) until CMV DNA levels reached their peak was significantly higher (*p* < 0.001) in CsCMVi episodes than in non‐CsCMVi episodes. cmvIL‐10 AUC between Days 14 and 23 after allo‐HCT (AUC₁₄₋₂₃) values were significantly higher in CsCMVi episodes compared with non‐CsCMVi episodes among patients receiving LMV therapy (*p* = 0.008). An AUC₁₄₋₂₃ cutoff value of log_10_ 3.06 discriminated anticipately between CsCMVi and non‐CsCMVi with a sensitivity and specificity of 100%. Plasma cmvIL‐10 levels may reflect true CMV replication and thus provide a unique perspective on viral dynamics, serving as an ancillary marker to CMV DNA monitoring.

## 1 Introduction

Monitoring CMV DNA load in blood is a cornerstone of cytomegalovirus (CMV) infection management in allogeneic hematopoietic transplant recipients (allo‐HCT) recipients, as it enables timely initiation of antiviral therapy to control viral replication and thereby minimize the risk of CMV end‐organ disease [1]. However, the presence of CMV DNA in blood does not always indicate an ongoing episode of viral replication, since free viral DNA may transiently leak into the bloodstream and be rapidly cleared (i.e., “blips” or self‐resolving episodes). This phenomenon is particularly frequent among patients receiving letermovir (LMV) prophylaxis [2, 3], as LMV inhibits the production of infectious viral particles but does not block CMV DNA replication [4]. While the kinetic pattern of CMV DNA in plasma or whole blood often provides valuable information in this context [5, 6], novel biomarkers of active CMV infection, such as CMV UL25.1 RNAemia, have been proposed [7, 8, 9]. The CMV gene *UL111a* encodes a homolog of human interleukin‐10 (hIL‐10), referred to as cmvIL‐10, which is predominantly expressed during lytic infection [10]. cmvIL‐10 exerts a broad range of immunosuppressive functions through its interaction with the human IL‐10 receptor (IL‐10R1) [11, 12]. Deep sequencing of the *UL111a* gene directly from clinical samples has revealed the presence of viral variants that may differentially modulate host immune responses [13]. Furthermore, it was recently shown that expression of cmvIL‐10 RNA in peripheral blood from kidney transplant recipients was positively associated with an increase in viral DNA detection in subsequent specimens, suggesting that monitoring cmvIL‐10 may be ancillary to viral DNA to allow early detection of active CMV infection in transplant recipients [14]. In this study, we investigated whether plasma cmvIL‐10 levels could serve as a biomarker of active CMV replication in patients with CMV DNAemia, occurring either during or outside LMV prophylaxis.

## 2 Materials and Methods

### 2.1 Patients and Specimens

A convenience panel of 189 leftover plasma samples that tested positive for CMV DNA using the Alinity *m* CMV assay (Abbott Molecular Inc., Des Plaines, IL, USA) [5] was assembled. These samples represented 33 episodes of CMV DNAemia occurring in unique CMV‐seropositive allo‐HCT recipients that underwent primary LMV prophylaxis (Table 1). Eighteen episodes developed during LMV prophylaxis (*n* = 110 specimens), and 15 occurred outside LMV prophylaxis (*n* = 79 specimens). Sixteen episodes of CMV DNAemia were classified as clinically significant (CsCMVi), defined as those in which peak CMV DNA levels exceeded the institutional threshold for preemptive antiviral therapy (1500 IU/mL, regardless of LMV prophylaxis status). Seven of these 16 CsCMVi episodes occurred in patients receiving LMV therapy. No CsCMVi episodes involved CMV end‐organ disease. All plasma samples had been cryopreserved at −80°C within 24 h of collection between January 2023 and February 2025 and had not been thawed before cmvIL‐10 testing. The current study was approved by the Institutional Review Board (IRB) Research Ethics Committee of Hospital Clínico Universitario INCLIVA (2024/153). The IRB issued an informed consent waiver.

**Table 1:** Demographic and clinical characteristics of the study population.

Parameter	Values
Male, no. (%)	18 (54.5)
Age (years), median (IQR)	57.9 (24–76)
Underlying hematological disease, no. (%)	
Acute myeloid leukemia	12 (36.4)
Chronic myeloid leukemia	5 (15.1)
Non‐Hodgkin lymphoma	5 (15.1)
Myelodysplastic syndrome	4 (12.1)
Hodgkin lymphoma	2 (6.1)
Acute lymphoblastic leukemia	2 (6.1)
T‐cell lymphoma	2 (6.1)
Myelofibrosis	1 (3)
Donor type, no. (%)	
Haploidentical	20 (60.6)
HLA‐matched unrelated donor	8 (24.2)
HLA‐mismatched unrelated donor	3 (9.1)
HLA‐matched related donor	2 (6.1)
CMV serostatus, no. (%)	
D+/R+	25 (75.7)
D−/R+	8 (24.3)
Conditioning regimen, no. (%)	
Myeloablative	2 (6.1)
Non‐myeloablative	31 (93.9)
GvHD prophylaxis, no. (%)	
Cyclophosphamide, sirolimus, and mycophenolate mofetil	33 (100)

Abbreviations: CMV, cytomegalovirus; D, donor; HLA, human leukocyte antigen; IQR, interquartile range; R, recipient.

### 2.2 Quantification of cmvIL‐10 in Plasma

Quantification of cmvIL‐10 in plasma was performed using an enzyme‐linked immunosorbent assay (ELISA) as previously described [15]. Briefly, 96‐well Nunc MaxiSorp plates were coated with 100 μL of capture antibody (cmvIL‐10 Affinity Purified Polyclonal Antibody, Goat IgG; R&D Systems, Minneapolis, MN) at a final concentration of 2 μg/mL and incubated overnight at 4°C. Following removal of the capture antibody, plates were washed three times with wash buffer (1 × PBS + 0.05% Tween 20) and blocked with blocking buffer (PBS + 3% bovine serum albumin) for 1 h at room temperature. After three additional washes, 100 μL of plasma samples (diluted 1:5) and standards were added in duplicate and incubated for 2 h at room temperature. Protein standards were prepared by serial twofold dilutions of recombinant cmvIL‐10 (R&D Systems) in protein standard dilution buffer (PBS + 0.1% BSA), ranging from 1000 to 15.625 pg/mL. Following incubation and washing, detection antibody (cmvIL‐10 Biotinylated Affinity Purified Polyclonal Antibody, Goat IgG; R&D Systems) was added at a final concentration of 0.2 μg/mL and incubated for 2 h at room temperature. After three washes, streptavidin‐HRP (R&D Systems) was added at working concentration and incubated for 20 min in the dark. Following three final washes, substrate solution (R&D Systems) was added and incubated in the dark for 20 min. The reaction was stopped with 50 μL of stop solution (1 M H₂SO₄), and optical density was measured at 450 nm using a Virclia instrument (Vircell, Granada, Spain). Standard curves were generated using GraphPad Prism software by plotting optical density values against recombinant cmvIL‐10 concentrations using four‐parameter logistic curve fitting. Plasma cmvIL‐10 concentrations (pg/mL) were interpolated from the standard curve.

### 2.3 Statistical Analyses

Differences between medians were compared using the Mann–Whitney *U* test. The degree of correlation between continuous variables was analyzed using Spearman's Rank test. The cmvIL‐10 area under the curve (AUC) was calculated when appropriate, and required two or more specimens/patient. The Youden index was used to determine the optimal AUC threshold to maximize the difference between the true positive rate (sensitivity) and the false positive rate (1‐specificity). Two‐sided exact *p*‐values are reported. A *p*‐value < 0.05 was considered statistically significant. The analyses were performed using the GraphPad Prism 9.0.2 statistical package.

## 3 Results

### 3.1 Correlation Between cmvIL‐10 and CMV DNA Levels in Plasma

**Figure 1:**
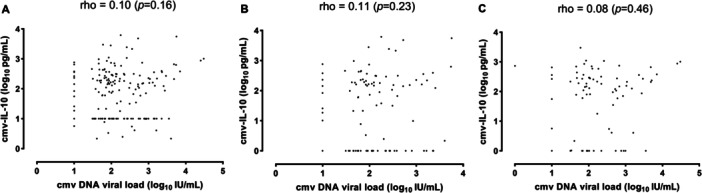
Correlation between cytomegalovirus (CMV) IL‐10 (cmvIL‐10) and CMV DNA levels in plasma from allogeneic hematopoietic stem cell transplant recipients. (A) All specimens; (B) specimens collected while patients were under letermovir prophylaxis; and (C) specimens collected while patients were not under letermovir prophylaxis. Spearman rank (Rho) and *p*‐values are shown.

We first investigated whether plasma cmvIL‐10 levels correlated with CMV DNA loads as measured by a real‐time PCR assay. A total of 189 plasma specimens with quantifiable CMV DNA, representing 33 episodes of active CMV infection that developed a median of 26 days after allo‐HCT (IQR, 8.5–65), were available for cmvIL‐10 testing (median of 5 samples per episode; range, 3–12). The timing of specimen collection relative to allo‐HCT is presented in Table S1. Among the 189 specimens, 135 tested positive for cmvIL‐10 (median value, 190 pg/mL; IQR, 82.3–373.2), while 54 were undetectable. Median cmvIL‐10 peak value was 396.5 pg/mL (range, 83–5657). As shown in Figure 1, there was an overall very weak correlation between the two biomarkers (Rho = 0.10; *p* = 0.16). The degree of correlation was poor regardless of whether patients were under LMV prophylaxis (Figure 1B) or not (Figure 1C).

### 3.2 cmvIL‐10 as a Marker of Active Infection

**Figure 2:**
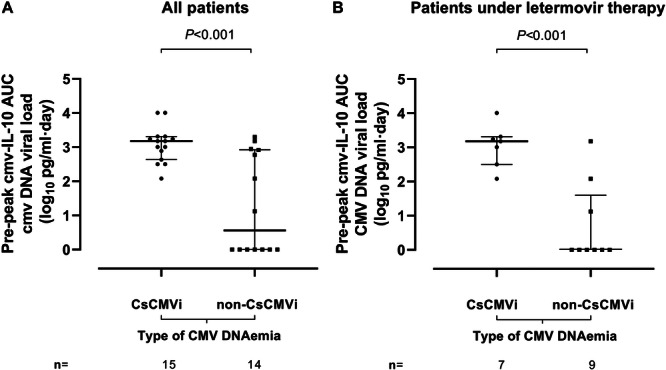
Area under the curve (AUC) of cytomegalovirus (CMV) IL‐10 levels (cmvIL‐10) measured in plasma up to the CMV DNA peak in allogeneic hematopoietic stem cell transplant recipients who either developed or did not develop clinically significant CMV infection (CsCMVi). (A) All patients in the cohort. (B) Patients receiving letermovir therapy at the time of CMV DNAemia occurrence. Bars represent medians and interquartile ranges. *p*‐Values for group comparisons are shown.

To assess whether cmvIL‐10 measurement could identify CsCMVi episodes, we calculated the AUCs using cmvIL‐10 values from specimens collected until the CMV DNA peak level. This parameter was available for 29 of the 33 episodes (CsCMVi, *n* = 15; non‐CsCMVi, *n* = 14). As shown in Figure 2A, the median cmvIL‐10 AUC was significantly higher (*p* < 0.001) in CsCMVi episodes than in non‐CsCMVi episodes (log_10_ 3.1 vs. log_10_ 0.56). Notably, the number of specimens used for AUC calculations was comparable across groups (median, three specimens; *p* ≥ 0.5), as was the timing of the first specimen collection after allo‐HCT (median, 18 days for CsCMVi vs. 19 days for non‐CsCMVi). A similar pattern was observed when only episodes occurring during LMV prophylaxis were analyzed separately (Figure 2B).

**Figure 3:**
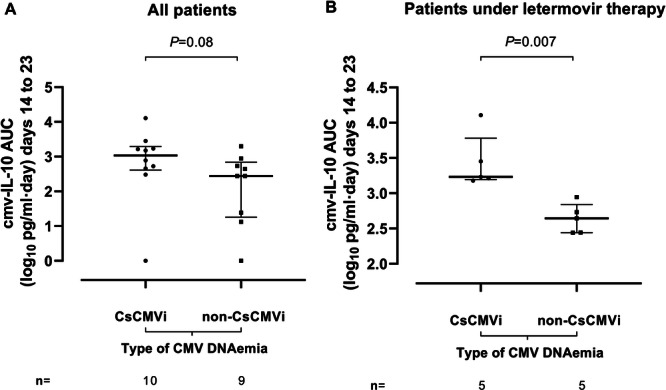
Area under the curve (AUC) of cytomegalovirus (CMV) IL‐10 levels (cmvIL‐10) measured in plasma between Days 14 and 23 (AUC₁₄₋₂₃) after allogeneic hematopoietic stem cell transplant in patients who either developed or did not develop clinically significant CMV infection (CsCMVi). (A) All patients in the cohort. (B) Patients receiving letermovir therapy at the time of CMV DNAemia occurrence. Bars represent medians and interquartile ranges. *p*‐Values for group comparisons are shown.

We next investigated whether cmvIL‐10 AUCs could predict the occurrence of CsCMVi. To this end, we calculated AUCs between days 14 and 23 post–allo‐HCT (cmvIL‐10 AUC₁₄₋₂₃), encompassing measurements from plasma specimens collected prior to the CMV DNAemia peak (median, 7 days; range, 6–10 days earlier). As shown in Figure 3A, there was a trend toward higher cmvIL‐10 AUC₁₄₋₂₃ values in CsCMVi episodes (median, log_10_ 3.03) compared with non‐CsCMVi episodes (median, log_10_ 2.4; *p* = 0.08). The median number of specimens used for AUC calculations was similar across groups (*n* = 3). In this setting, an AUC cutoff value of log_10_ 2.45 provided the most efficient discrimination between CsCMVi and non‐CsCMVi, with a sensitivity of 50% and a specificity of 89% (AUC = 0.73; *p* = 0.06). Interestingly, cmvIL‐10 AUC₁₄₋₂₃ values were significantly higher in CsCMVi episodes compared with non‐CsCMVi episodes among patients receiving LMV therapy (*p* = 0.008). In this setting, an AUC₁₄₋₂₃ cutoff value of log_10_ 3.06 discriminated perfectly across comparison groups, with a sensitivity and specificity of 100% (AUC = 1; *p* = 0.009).

## 4 Discussion

The kinetics of CMV DNA in blood has proven to be a useful parameter for anticipating the occurrence of clinically significant CMV infection (CsCMVi) in allo‐HCT recipients irrespective of the real‐time PCR assay employed [16, 17]. It is well documented that the presence of CMV DNA in blood may not necessarily reflect active CMV infection in tissues or in the blood compartment due to the mechanism of action of LMV [2, 3]. In this context, identifying episodes of CMV DNAemia that represent true viral replication, as opposed to abortive infection, is particularly relevant in patients receiving LMV, to avoid unnecessary interruption of therapy. Our group has demonstrated the potential value of CMV DNA doubling time (CMV dt) for the early identification of true episodes of active CMV infection, both in the presence and absence of LMV treatment [5, 6, 16, 17]; nevertheless, the clinical performance of this parameter, in terms of its predictive value, is not maximally accurate.

Monitoring of CMV UL25.1 RNAemia using a commercially available assay has been proposed as a reliable marker of active CMV infection, since this late CMV transcript appears to be virion‐associated in plasma [7, 8]. Nevertheless, because LMV does not inhibit either CMV DNA replication or the synthesis of late CMV mRNAs, we believe that this marker does not substantially enhance the information already provided by quantitative CMV DNA testing in patients receiving LMV [9]. Here, we reasoned that monitoring plasma cmvIL‐10 levels might aid in the early identification of CsCMVi, as this viral human‐homolog cytokine is mainly synthesized during viral replication and exerts immunosuppressive effects that may vary across strains [10, 11, 12, 13]. We found a negligible correlation between CMV DNA and cmvIL‐10 levels, in contrast to what has been reported for CMV UL25.1 RNAemia. Although speculative, this observation may indicate that the detection of cmvIL‐10 in blood at certain levels more accurately reflects the presence of infectious viral particles in the bloodstream as a result of productive infection in tissues or even within the blood compartment, compared with the detection of particle‐free viral DNA in plasma. Furthermore, monitoring cmvIL‐10 levels could reliably identify and, more importantly, anticipate clinically significant CMV infection (CsCMVi) both in the presence and absence of LMV therapy. Notably, an AUC cutoff value of log₁₀ 3.06 perfectly discriminated between CsCMVi and non‐CsCMVi groups, with this distinction occurring a median of 1 week before CMV DNAemia reached levels sufficient for classification as such. We acknowledge two main limitations of this study: first, the very small sample size, and second, the lack of CMV end‐organ disease cases among CsCMVi episodes. Taken together, our findings support the notion that plasma cmvIL‐10 levels may reflect true CMV replication and thus provide a unique perspective on viral dynamics, serving as an ancillary marker to CMV DNA monitoring. Nevertheless, well‐designed prospective studies are warranted to further assess the clinical utility of cnvIL‐10 testing, particularly in LMV‐treated patients.

## Author Contributions

A.S.‐S., E.A., E.G., E.C., A.P., J.L.P., and C.S., data curation and analysis, methodology. D.N, conceptualization, supervision, data analysis and writing.

## Ethics Statement

The current study was approved by the Institutional Review Board (IRB) Research Ethics Committee of Hospital Clínico Universitario INCLIVA (2024/153). The IRB issued an informed consent waiver.

## Conflicts of Interest

J.L.P., C.S., and D.N. received honoraria for conferences sponsored by MSD.

## Supporting information


**Supplementary Table 1:** Available plasma specimen for Cytomegalovirus Interleukin‐10 homologue testing.

## Data Availability

The data that support the findings of this study are available in the Supporting Information of this article. The datasets generated and/or analyzed during the current study are available from the corresponding author upon reasonable request.
