# Supplementary material for: Cytomegalovirus IL‐10 in Plasma as a Marker of Active Infection in Allogeneic Hematopoietic Transplant Recipients: An Exploratory Study
Source: J Med Virol. 2026 Jan 13;98(1):e70806. doi: 10.1002/jmv.70806 (PMC12797184; doi:10.1002/jmv.70806)
Supplement: Supplementary file 1 — Supplementary Table 1: Available plasma specimen for Cytomegalovirus Interleukin‐10 homologue testing. [file JMV-98-e70806-s001.docx]

| **Supplementary Table 1. Available plasma specimen for Cytomegalovirus Interleukin-10 homologue testing** | | | | |
| --- | --- | --- | --- | --- |
| **Patient number** | **Undergoing Letermovir prophylaxis at the time of testing** | **Day of first measurement of cmvIL-10 after allo-HCT** | **Day of last measurement of cmvIL-10 after allo-HCT** | **No. of determinations** |
| 1 | No | 18 | 70 | 5 |
| 2 | No | 45 | 84 | 4 |
| 3 | No | 15 | 101 | 6 |
| 4 | No | 14 | 67 | 6 |
| 5 | No | 22 | 124 | 5 |
| 6 | No | 136 | 212 | 5 |
| 7 | No | 20 | 77 | 5 |
| 8 | No | 21 | 83 | 4 |
| 9 | No | 108 | 157 | 4 |
| 10 | No | 19 | 100 | 6 |
| 11 | No | 23 | 56 | 5 |
| 12 | No | 160 | 223 | 6 |
| 13 | No | 14 | 54 | 9 |
| 14 | No | 134 | 181 | 6 |
| 15 | No | 250 | 278 | 6 |
| 16 | Yes | 100 | 228 | 4 |
| 17 | Yes | 7 | 72 | 8 |
| 18 | Yes^a^ | 271 | 315 | 5 |
| 19 | Yes | 6 | 15 | 3 |
| 20 | Yes | 12 | 186 | 12 |
| 21 | Yes | 10 | 62 | 7 |
| 22 | Yes | 4 | 50 | 8 |
| 23 | Yes | 33 | 61 | 3 |
| 24 | Yes | 12 | 58 | 5 |
| 25 | Yes | 5 | 23 | 5 |
| 26 | Yes | 35 | 66 | 6 |
| 27 | Yes | 6 | 20 | 4 |
| 28 | Yes | 79 | 105 | 5 |
| 29 | Yes | 7 | 43 | 8 |
| 30 | Yes | 33 | 89 | 5 |
| 31 | Yes | 106 | 138 | 4 |
| 33 | Yes | 4 | 39 | 8 |
| 33 | Yes | 38 | 143 | 7 |
| Allo-HCT, allogeneic hematopoietic stem cell transplantation; cmvIL10, Cytomegalovirus Interleukin-10 homologue  ^a^Secondary prophylaxis | | | | |
